# Annual Commencement of Columbia Veterinary College and School of Comparative Medicine

**Published:** 1883-04

**Authors:** 


### Reports of Commencements and Societies.

### ANNUAL COMMENCEMENT OF COLUMBIA VETERINARY COLLEGE AND SCHOOL OF COMPARATIVE MEDICINE.

The closing exercises of the above institution took place in Chickering Hall, Thursday evening, March 29th. The hall was crowded to its utmost capacity with a very enthusiastic and intelligent audience. The stage was elaborately and handsomely decorated with a profusion of tropical plants, the Faculty, Trustees and invited guests occupying the seats, and forming a substantial background for the interesting exercises which followed. The graduating class, twenty-five in number, filled the first seats in the auditorium, The degree of Doctor of Veterinary Science was conferred by Alex. Hadden, M.D., President of the Board of Trustees. In making his final charge to the graduates he complimented the class on the high rank they had manifested on final examination, and said with these "Presents:"

"Gentlemen, you may enter upon the active duties of the profession of your choice with the full assurance that you are entitled to all the rights and privileges vouchsafed to you by the statutory laws of the Empire State. Inseparably connected with such privileges is the blessing of your Alma Mater. Her benign influences will follow you wherever you may go. She will watch your professional career with peculiar interest. Her jealous eye will delight in your prosperity, and triumph in every advancement you may make in the fields of science which are now opened out before you. She will be ambitious to have you occupy the loftiest plains of practical science, and will rejoice in all that makes your pathways pleasant in life. In adversity your misfortunes and ills will be to her a burden and a cause of grief. We now commit you to the public, and ask her to receive you kindly, not as strangers or enemies, but as friends and ministers of mercy in whom implicit confidence may be placed, believing that all the duties which may fall to your lot in the special departments of Comparative Medical and Surgical Science may be intelligently, earnestly and honorably performed, and knowing that sanitarians and practitioners of human medicine can look to you without disappointment for aid and information in investigations highly important to the health and well being of mankind."

The Honor men of the Junior Class were then awarded the Junior Certificate of Honor by Prof. E. S. Bates, Dean of the Faculty.

Prof. Hubbard W. Mitchell awarded the prizes as follows:

R. A. Kœmpel, Ph.D.D.V.S., of New York, Faculty Gold Medal.

T. E. Finnegan, D.V.S., of New York, Alumni Anatomical Gold Medal.

Henry Spencer, of Junior Class, Alumni Laboratory Gold Medal.

W. H. Gribble, of Junior Class, Faculty Prize Silver Medal.

J. A. Soule, D.V.S., Silver Medal, Shoeing Prize.

W. E. A. Cuff, D.V.S., Hawks' Essay Prize, Gold Medal.

Wm. Herbert Lowe, D.V.S., Columbia Clinic Prize, Case of Instruments.

J. R. Cosgrove, D.V.S., Jurisprudence Prize, Case of Instruments.

W. H. Gribble, Junior Student, General Anatomical Prize, Case of Instruments.

F. A. K. O'Shea, Chemistry Prize, Case of Instruments.

W. H. Gribble, Robertson Prize.

R. L. Parkin, D.V.S., Clinical Cattle Prize.

Adolph. Vollmiers, Pathological Prize.

H. C. Slee, D.V.S., Physiology Prize.

Chas. B. Uibel, Wm. R. Jenkins' Prize, for Specimens of Taxidermy.

The valedictory address was delivered by H. C Slee, D.V.S., of the graduating class. It was marked for its exceeding originality, conciseness of expression, and the masterly style in which it was pronounced.

H. L. Ramacciotto, D.V.S., made the hit of the evening with his recitation of "Flash, the Fireman's Story."

Addresses were delivered to the graduates by Judge W. E. Curtis and Hon. Andrew L. Soulard.

The musical contributions were peculiarily excellent. The orchestra, under the direction of Louis Eckert, were honored with several encores. The "Third Aria Varia," rendered by the Stang Bros., as well as the cornet solos by Sig. Liberati, were artistically rendered and added much to the pleasure of the occasion.

The very pleasant and interesting commencement programme closed the most satisfactory time which has thus far marked the history of the College. The number of students in attendance has been larger than could be comfortably accommodated. The classes manifesting unusual interest in the various subjects to which their attention was called, and on final examination showing greater proficiency and more thorough training than any class which has yet graduated from the College.

The Trustees and Faculty realizing the inadequacy of the present accommodations have purchased a new site so that the next term will probably open in new buildings with greatly increased facilities for doing the work to which the College is dedicated.

The history of thc college is markedly progressive. From small beginnings and small classes it has, within the short space of five years, attained the reputation of being the best College in the country devoted to the study of comparative medicine and surgery. Its matriculants outnumbering this last session those of any similar institution in the United States.

LIST OF GRADUATING CLASS

Asa Redington Balkam, D.V.S., Lewiston Maine.
James Robert Cosgrove, D.V.S., New York.
Emilio Charum, D.V.S., New York.
Fred. Cooper Curtice, D.V.S., Moravia, N. Y.

William Emmet Aloysius Cuff, D.V.S., New York.
Theodore De Clyne, D.V.S., New Durham, N. J.
Thomas Edward Finnegan, D.V.S., New York.
Theodore F. Hance, M.D., D.V.S., Newark, N. J.
Walter Hugheson Jackson, D.V.S., Poughkeepsie, N. Y.
Robert Augustus Kœmple. Ph.D.D.V.S., New York.
Thomas S. Lippincott, D.V.S., Burlington, N. J.
William Herbert Lowe, D.V.S., Little Falls, N. J.
Robert Lincoln Parkin, D.V.S., Brooklyn, N. Y.
Hugo Louis Ramacciotto, D.V.S., New York.
Henry Whitfield Rowland, D.V.S., Metuchen, N. J.
William Henry Shaw, D.V.S., Roscoe, Ill.
Benjamin Herbert Swan, Ph.D.D.V.S., Chesterfield Factory, N. H.
John Albion Soule, D.V.S., Hyde Park, Mass.
Albert D. Sturges, D.V.S., Wilton, Conn.
Robert William Stuart, D.V.S., Mt. Victory, Ohio.
Frederick Emil Schultze, D.V.S., Brooklyn, N. Y.
Henry Child Slee, D.V.S., Brooklyn, N. Y.
Paul Will, D.V.S., Jersey City, N. J.
R. Trall Whittlesey, D.V.S., Emporia, Kansas.
Maximilian Weise, D.V.S., New York.

LIST OF HONOR MEN, JUNIOR CLASS.

W. H. Gribble, Churchville, N. Y.
Chas. B. Uibel, Reamstown, Pa.
John Hamlin, Afton, N. Y.
E. A. Parsons, Hartford, Conn.
B. A. Plint, Jr., New York City.
R. A. Stoute, Barbadoes, W. I.
W. H. Mook, Metuchen, N. J.
Charles Kuehner, Jersey City N. J.
J. L. Windolph, Darlington, Md.
Adolph. Vollmiers, New York City.

